# Factores asociados a la incidencia de casos de tuberculosis en el municipio de Riohacha, 2018-2022

**DOI:** 10.15446/rsap.V25n3.110829

**Published:** 2023-05-01

**Authors:** Diana C. Molina Ariza, Andrés Felipe Aponte-Gutiérrez, Jesús A. Berdugo-Gutiérrez

**Affiliations:** 1 DM: Bact. M. Sc. Salud Pública. Universidad Nacional de Colombia, Sede La Paz. Valledupar. Cesar, Colombia. dimolinaa@unal.edu.co Universidad Nacional de Colombia Salud Pública Universidad Nacional de Colombia Valledupar Cesar Colombia dimolinaa@unal.edu.co; 2 AA: Biólogo, M. Sc. Ciencias Biológicas. Grupo de Investigación en Ciencias de la Orinoquía, Universidad Nacional de Colombia. Orinoquia, Colombia. anaponteg@unal.edu.co Universidad Nacional de Colombia Ciencias Biológicas Grupo de Investigación en Ciencias de la Orinoquía Universidad Nacional de Colombia Orinoquia Colombia anaponteg@unal.edu.co; 3 JB: MV. M. Sc. Genética Humana Ph.D. Ciencias Agrarias. Universidad Nacional de Colombia. Orinoquia. Colombia. jaberdugog@unal.edu.co Universidad Nacional de Colombia Ciencias Agrarias Universidad Nacional de Colombia Orinoquia Colombia jaberdugog@unal.edu.co

**Keywords:** Tuberculosis, Riohacha, migración *(fuente: DeCS, BIREME)*, Tuberculosis, Riohacha, migration *(source: MeSH, NLM)*

## Abstract

**Objetivo:**

La tuberculosis, enfermedad curable y prevenible, es una de las diez primeras causas de morbimortalidad en la población mundial. Según los boletines epidemiológicos de la Alcaldía de Riohacha, se observa un aumento inexplicable de casos de la enfermedad en los últimos cinco años.

**Métodos:**

Este trabajo analiza los factores asociados a la incidencia de casos de tuberculosis desde el 2018 al 2022 notificados en el Sivigila. Fueron analizadas diferentes variables entre los años mediante la comparación de proporciones y en conjunto con un análisis de componentes principales usando la plataforma R.

**Resultados:**

La enfermedad se presentó con mayor frecuencia en hombres y en personas sin seguridad social. La distribución etárea fue la misma en los años evaluados; en relación con la pertenencia étnica, el 90 °% de los positivos fueron indígenas o migrantes. La ocupación fue la variable que mayormente explica la presentación de los casos; ama de casa y "no aplica" fueron las más importantes (48 °%). No hay diferencias en la forma de diagnóstico en los casos evaluados.

**Conclusiones:**

Se concluye que la migración y la falta de seguridad social contribuyen al aumento de los casos de tuberculosis observados. Es paradójico que siendo los hombres los más afectados, la ocupación con más frecuencia de presentación fue ama de casa.

## Abstract (en)

**Objective:**

Tuberculosis, a curable and preventable disease, persists as one of the 10 leading causes of morbidity and mortality in the world population. According to epidemiological bulletins from the Mayor's Office of Riohacha, there has been an unexplained increase in the number of cases in the last five years.

**Methods:**

This paper analyzes the factors associated with the incidence of tuberculosis cases from 2018 to 2022 reported in Sivigila. Different variables were analyzed and compared by comparison of proportions especially between 2021, 2021 and 2022 and as a group analyzed by principal component analysis using the R platform.

**Results:**

It was found that men had a higher presentation than women, and of people without social security, that the age distribution of the disease was the same in the years evaluated, 90 °% of the positives were indigenous or migrants with a significant increase in the number of migrants. Occupation was the variable that most explained the presentation of the cases, and among them housewives and "not applicable" were the most important (48 °%), and there were no differences in the form of diagnosis in all evaluated.

**Conclusions:**

It can be concluded that migration and the lack of social security contribute to the increase in the number of tuberculosis cases observed. It is paradoxical that men are the most affected but that the major occupation of presentation is housewives.

La tuberculosis es una enfermedad curable y prevenible, causada por el *Mycobacterium tuberculosis,* una bacteria intracelular, cuyo órgano blanco es casi siempre los pulmones. El microorganismo puede viajar a través del aire, lo que facilita su transmisión; si un infectado tose, estornuda o escupe, irremediablemente expulsará bacilos tuberculosos que las personas a su alrededor pueden inhalar y así infectarse 1. Los signos y los síntomas que tiene un infectado son: tos y expectoración, dolor torácico, pérdida de peso, hemoptisis, falta de apetito, astenia, fiebre, escalofríos y sudores nocturnos. Algunas personas presentan enfermedad pulmonar poco después de contraer la infección y otras pueden enfermar años después, cuando su sistema inmunitario se debilita 2.

En las personas con deficiencias en su respuesta inmune, especialmente infectadas por el virus de la inmunodeficiencia humana (VIH), el riesgo de presentar la enfermedad es más alto que para las personas con el sistema inmunitario normal, lo que genera un patrón de susceptibilidad. También existen algunas condiciones que favorecen la presentación de la enfermedad, como, por ejemplo, contactos cercanos con personas afectadas por tuberculosis; los migrantes, especialmente aquellos que vienen de regiones del mundo con altas tasas de tuberculosis; niños menores de cinco años positivos para la enfermedad; personas que viven en la calle; usuarios de drogas inyectables e infectados con VIH 3.

Las desigualdades socioeconómicas, la migración, el crecimiento rápido de la población, la falta de alimentos, la malnutrición, las malas condiciones ambientales y de vivienda, las barreras geográficas y de acceso a la salud son determinantes que pueden permitir el desarrollo de la tuberculosis (TB).

Factores como la clase social, la educación, la ocupación, la pertenencia étnica y el ingreso impactan en el desarrollo de la TB, incluso antes de enfermar. Con el fin de estimar el riesgo atribuible a las poblaciones, la Organización Mundial de la Salud (OMS) hizo un análisis de los factores que en los diferentes países aportan mayor carga de TB a nivel global, y se concluyó que la presencia de infección por VIH, la malnutrición, el hábito tabáquico, la diabetes, el abuso del alcohol y la contaminación intradomiciliaria eran los determinantes que mayormente contribuían al riesgo poblacional de TB. Coincidentemente, dichos factores son más frecuentes en los grupos de nivel socioeconómico bajo, quienes además tienen mayor probabilidad de tener contacto con personas con TB activa y en ambientes que favorecen la infección 4.

A pesar de los avances científicos y tecnológicos en el diagnóstico y el tratamiento de la TB, esta enfermedad persiste como una de las 10 primeras causas de morbimortalidad en la población mundial, aunque de acuerdo con los últimos datos del informe global de tuberculosis de la OMS, en el año 2020 hubo una disminución en el reporte de casos de tuberculosis en el mundo, con incremento en los fallecimientos y dificultades para el seguimiento al tratamiento, como consecuencia directa del impacto de la COVID-19, lo cual hizo que se incrementara nuevamente la brecha en relación con el acceso a los sistemas de salud y las condiciones de vida a nivel social y económico 5.

En el municipio de Riohacha, en el 2018 había una población wayúu de 380 460 individuos, con un crecimiento del 40,7 % en los últimos 13 años y una tasa de crecimiento anual del 2,6 % 6.

Según informaciones demográficas del departamento de La Guajira, debido a la emergencia sanitaria por el COVID-19, entre enero del 2019 y el 2020 la población venezolana en el departamento había aumentado en más de 20 000 personas; también al 3 de mayo del 2021, más de 12 200 personas retornaron a Venezuela. La Guajira es el quinto departamento con mayor población venezolana en Colombia, con 150 806 personas distribuidas en los 15 municipios; el 56,4 % de esta población tiene un estatus migratorio irregular. Los municipios con mayor concentración de venezolanos son Maicao (51 361) y Riohacha (47 172), que reúnen al 65 % del total; adicionalmente, el 22,3 % de los refugiados y migrantes de Venezuela en el departamento son niños, niñas y adolescentes; y el 3,3 % son adultos mayores. Según Migración Colombia, entre enero y julio del 2022, en el departamento de La Guajira se registraron 5 131 entradas de personas refugiadas y migrantes venezolanas, siendo Maicao (3 415) y Riohacha (1 572) las ciudades que tienen un mayor índice de entradas, con un total de 4 987 refugiados/ migrantes venezolanos 7.

El análisis de componentes principales (ACP) es una metodología estadística para establecer preliminarmente los factores que interfieren en la variabilidad de los datos de un conjunto de información multiespacial 8. El objetivo del grupo investigador fue conocer los factores asociados a la incidencia de casos de tuberculosis en el municipio de Riohacha, entre los año 2018 y 2022, para aportar al conocimiento de la epidemiología de le enfermedad en la región y tratar de comprender el aumento de su presencia, usando para tal fin la información que se registran en el Sistema de Vigilancia en Salud Pública (Sivigila).

## METODOLOGÍA

Se realizó un análisis descriptivo transversal de los casos registrados de tuberculosis pulmonar durante los años 2018 a 2022 en la ciudad de Riohacha, Colombia, registrado en el portal Sivigila del Instituto Nacional de Salud. Para ello, se tomaron los microdatos del código 820 sobre tuberculosis pulmonar.

Para el análisis, las variables se agruparon en las siguientes dimensiones: demográficas (sexo, edad, pertenencia étnica, tipo de régimen, ocupación) y, adicionalmente, el tipo de diagnóstico. Se analizó el comportamiento de los cuatro años.

La información se depositó en una hoja de cálculo y se agrupó de acuerdo con las variables. Se hizo un recuento de la información, para lo cual se calculó el número de casos y las proporciones de presentación de cada una de ellas, que fueron comparadas mediante la comparación de proporciones con el paquete R; se consideró significativo un valor p < 0,05. También se utilizó un análisis de componentes principales (ACP) para establecer las variables que interfieren en la presentación de los datos en un conjunto de información multiespacial 8, para buscar la variable que pudiera explicar mejor el aumento observado en la presentación de los casos. Para algunas variables, en las cuales los estados se encuentran expresados de manera cualitativa, se realizó una transformación numérica; se consideró un estado como un valor ascendente iniciando desde 1 hasta el número final de variables.

### Consideraciones éticas

La información para llevar a cabo este estudio se obtuvo de la publicación de los datos de tuberculosis pulmonar en el portal del Sivigila, cuyos datos no revelan la identidad de las personas, lo que garantiza la confidencialidad de la información.

## RESULTADOS

**Figura 1:**
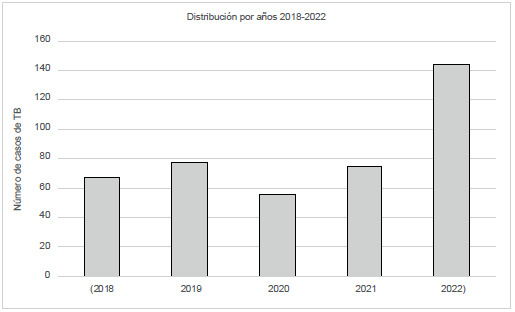
Presentación de casos de tuberculosis en Riohacha, 2018-2022

En el municipio de Riohacha, según el portal SIVIGILA del Instituto Nacional de Salud, durante el 2022 se notificaron 144 casos de tuberculosis; en el 2021 se notificaron 75 9, y se observó un incremento del 192 % para el 2022 (Figura 1), con una tasa de incidencia de 146 por 100 000 habitantes.

**Figura 2:**
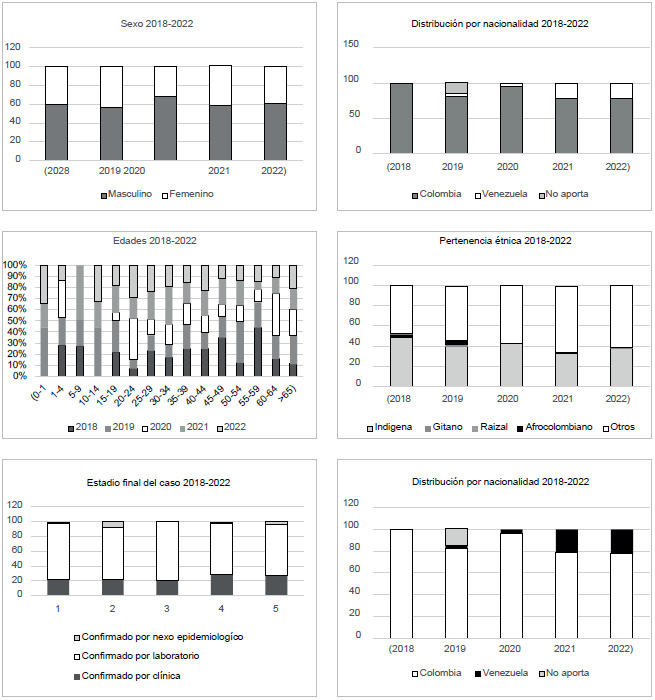
Resumen grafico de las variables analizadas durante los años 2018-2022

**Figura 3:**
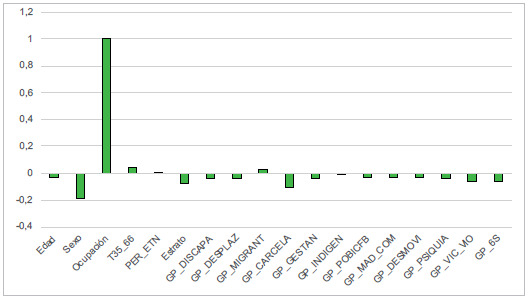
Representación gráfica del análisis de componentes principales de las variables registrads en la base de datos

A continuación se describen los resultados de las variables sociodemográficas analizadas, cuyas gráficas se presentan en conjunto en la Figura 2 y el ACP en la Figura 3.

### Sexo

Para el analisis de las variables sociodemográficas se puede observar que durante los años 2018, 2019, 2020, 2021 y 2022 el sexo masculino es el que mayor número de casos de TB presenta, con 60 % (año 2018), 56 % (año 2019), 68 % (año 2020), 59 % (año 2021) y 61 % (2022). Estas diferencias son significativas (p<0,05), con excepcion del 2019 (p=0,139).

### Edad

El grupo de etáreo que más afectado por TB en el año 2018 fue el de 25 a 29 años (14,9 %), seguido por el de 55 a 59 años (14,9 %). En el 2019 los mas afectados fueron los de 20 a 24 años (9,1 %) y los mayores de 65 años (16,9 %); en el 2020, los de 20 a 24 años (23,2 %) y los mayores de 65 años (16,1 %); en el año 2021, los de 25 a 29 años (16 %), seguidos de los mayores de 65 años (12 %, p<0,05). En el 2022, los de 20 a 24 años representaron el 18 % y los mayores de 65 años el 14 % (p<0,05).

Cuando se evalúa la edad como una viarable, no se encuentran diferencias significativas entre los grupos durante los años analizados; sin embargo, se observan diferencias estadísticamente significativas en los diferentes grupos etáreos analizados.

## Pertenencia étnica

Durante los años evaluados, los indígenas representaron el 80,6 % de los casos, seguidos de la población migrante (19,4 %). Estas diferencias fueron significativamente (p<0,05); otras pertenencias étnicas, como los raizales, son apenas mencionadas.

## Nacionalidad

Se pudo evidenciar que en el año 2018, el 100 % de los casos notificados eran de nacionalidad colombiana, en tanto que en el 2019 y en el 2020 se notificaron dos casos cada año con nacionalidad venezolana, y en el año 2021 se informan 16 casos (21 %). Para el 2022 se informan 22 casos (22,2 %) de personas afectadas con tuberculosis con esta última nacionalidad. En comparación con los cuatro casos del año 2020, los años 2021 y 2022 son estadísticamente significativos (p<0,05).

## Grupo poblacional

La población indígena estuvo afectada: se presentaron 33 casos en el año 2018, 31 en el 2019, 24 en el 2020, 25 en el 2021 y 54 en el 2022. Los habitantes de calle, los desplazados y las madres comunitarias hacen parte de la población afectada por tuberculosis en una proporción menor; en el año 2021 empezaron a notificarse los migrantes con 16 casos (21 %), en el 2022 se informaron 22 (28,7 %).

## Tipo de seguridad social

En el año 2018, la mayor proporción de presentación de TB se dio en el regimen subsidiado (75 %), mientras que en el 2019 correspondió al 82 %, en el 2020 al 80 %, en el 2021 al 65 % y en el 2022 al 63 %, con un descenso significativo comparado con los años anteriores al 2020 (p < 0,05). El grupo que siguió fueron los no asegurados, que en el 2021 correspondieron al 23 % y en el 2022 al 20 %, cifra superior a los años anteriores al 2020, con una diferencia estadísticamente significativa.

## Tipo de caso

Los casos confirmados por laboratorio en el año 2018 representaron un 76 %, en el 2019 un 69 %, en el 2020 un 73 %, en el 2021 un 75 %, y en el 2022 un 73 %. En los casos confirmados por clínica sobresale un 22 % a 29 % durante los años 2018 a 2022, sin que estas diferencias sean significativas.

## Ocupación

Las amas de casa son un grupo afectado, en el cual se presentaron 22 casos (32 %) en el 2018, 22 (28,6 %) en el 2019, 13 (23,2 %) en el 2020, 22 (29,3 %) en el 2021, 22, 29 (20,0 %) en el 2022. A este grupo le siguió el de "no aplica", que presentó 19 casos de tuberculosis en el año 2018 (28,4 %), 24 (31,2 %) en el 2019, 25 (44,6 %) en el 2020, 26 (34,7 %) en el 2021 y 71 (58,0 %) en el 2022. Tambien se presentaron casos en los estudiantes: 5 (7,5 %) en el 2018, 11 (14,3 %) en el 2019 y 6 (8 %) en el 2021. En el 2022 el otro grupo importante fue el de "trabajos varios" con 16 casos (11,1 %). El análisis de componentes principales mostró que este compentente explicaba el 99,9 % de la variación observada, siendo la variable *ocupación* la que representó la mayor fuente de variación entre los datos analizados. Para los autores, esta variable es la más importante dentro de la presentación de la enfermedad.

## DISCUSIÓN

En este trabajo se presenta por primera vez en la literatura un análsis correspondiente a la presentación de la tuberculosis en el municipio de Riohacha, entre el año 2018 y el 2022; en este último año se presentó un aumento de casos de tuberculosis, con una variacion del 192 % (62 casos) en comparación con el 2018. En las primeras 21 semanas del 2022 se presentó una incidencia de 28,47/100 000 habitantes, cifra mayor a la incidencia de tuberculosis a nivel nacional que es de 14,3/100 000 habitantes, lo que refleja un incremento de casos de TB en el municipio de Riohacha. Las mayores tasas de incidencia de tuberculosis del año 2022 se presentaron en Amazonas, Risaralda, Cali, Guaviare, Barranquilla y Caquetá; a estos aumentos de casos se puede agregar que la pandemia COVID-19 afectó la detección de casos de tuberculosis en los años anteriores al 2022 10.

Una vez analizadas las 16 variables asociadas con la presentación de TB, se encontró al sexo como una de las más importantes: el mayor número de casos se presentó en varones, posiblemente por las ocupaciones laborales o el ambiente social en el que se desenvuelven. Otro factor de riesgo es la edad: adultos de 25 a 34 años y mayores de 65 años; estos últimos se ven afectados por tener un sistema inmune con poca capacidad de respuesta y ser más susceptibles de adquirir la enfermedad. La pertenencia étnica es otro factor de riesgo: resalta que en el municipio de Riohacha habitan indígenas wayúu, un pueblo binacional que también reside en territorio venezolano; esta población indígena es un grupo muy vulnerable para adquirir la enfermedad debido a que en sus comunidades predominan factores como las condiciones de vivienda inadecuadas, sin servicios públicos, bajos ingresos económicos, malnutrición, hacinamiento, cocinar con leña, tabaquismo, consumo de alcohol; la mayoría de los indígenas viven en zonas rurales muy dispersas, en rancherías o caseríos, a varios minutos de camino, lo que se convierte en una barrera geográfica y dificulta el acceso a los servicios de salud de la persona afectada por tuberculosis; además, impide que consulte de manera oportuna y llega a ser un foco de infección para las personas que conviven con ella 11.

En el año 2018 no se notificaron casos de tuberculosis en venezolanos, sin embargo, en el 2021 se notificaron 16 (21 %). Se considera que esta observación pueda relacionarse con lo visto en la variable régimen de seguridad social no asegurados, que en el año 2021 presentó 17 casos, comparado con los tres casos del 2021, lo que convierte a la migración en un factor de riesgo, por lo menos para la región, y aumenta la incidencia de casos en el municipio de Riohacha.

En cuanto a la ocupación, las amas de casas son un grupo muy vulnerable. Teniendo en cuenta que la mayoría de los casos son hombres mayores de 65 años, se puede considerar que la fuente de infección para estas personas se encuentra en casa, dentro de las obligaciones para cuidar a los adultos. En el otro grupo afectado por tuberculosis, "No aplica", se encuentran las personas que no pueden trabajar, como los bebés, la población de la tercera edad. Al revisar la base de datos hay personas de todas las edades, por lo que se presenta un subregistro, y las UPGD notifica-doras no reportan la ocupación. La variable de ocupación es un dato muy importante que nos permite analizar la población laboral que se encuentra afectada por TB.

En un estudio de caracterización de la ocupación de pacientes con TB, y TB resistentes a múltiples medicamentos, en instituciones de tercer nivel en Bogotá, se encontró que el 26,1% se dedicaba al hogar, el 18,5% no tenía información en cuanto a la ocupación, el 8,3% eran militares y el 47,1% restante correspondía a otras ocupaciones, entre las que se resaltan estudiantes, vendedores ambulantes, campesinos, obreros y desempleados, y cabe destacar que el 2,2% de la muestra correspondía a trabajadores de la salud 12, resultados muy similares a los obtenidos en este análisis.

Se deben hacer esfuerzos para disminuir hasta cero aquellas ocupaciones que se registran en la encuesta como "no aplican", dada la alta proporción que presentan, lo que genera dificultad para la interpretación de los datos y la aplicación de medidas sanitarias en los lugares de trabajo. De esta manera, podría reducirse la infección por TB en el ámbito laboral.

Los trabajadores sanitarios tienen más riesgo que la población general de infectarse y enfermar por tuberculosis. El riesgo es mayor en los profesionales que tienen contacto con las secreciones de los pacientes, varía en cada país, e incluso entre regiones de un mismo país, posiblemente en función de la distinta aplicación de las medidas de control tanto en la comunidad como en los centros sanitarios 13; sin embargo, no tienen una participación importante en los resultados de este trabajo.

En el 2022 se observa que el grupo de riesgo según el sexo es el masculino; la población indigena continúa presentando casos de tuberculosis, y en cuanto al tipo de seguridad social, el mayor número de casos se presenta en el régimen subsidiado.

Si bien es cierto que la base de datos de tuberculosis se construye a partir de la información que las instituciones prestadoras de servicio de salud (IPS) recogen de cada uno de los pacientes que consultan, y esta información es registrada en el Sivigila de cada municipio, se pueden observar algunas limitaciones: se nota que en la variable ocupación habría un subregistro, evidenciado por la gran cantidad de registros "no aplica", dificultad en el registro del sexo y los grupos poblacionales donde hay muy pocos registros diferentes a los mencionados en los resultados, entre ellos la variable nexo epidemiológico.

En un estudio realizado sobre factores asociados a la prevalencia de tuberculosis en el distrito de Cartagena, se concluyó que la vacunación en los niños recién nacidos es un aspecto importante para la prevención de la enfermedad en la edad adulta, y se encontró una disminución del riesgo de padecer tuberculosis cuando la persona ha sido vacunada. El antecedente familiar es uno de los principales factores relacionados con la aparición de esta enfermedad, sumado a las medidas preventivas insuficientes dentro del núcleo familiar de la persona que vive con tuberculosis, lo que aumenta de forma considerable el riesgo de infección y progresión de la enfermedad 14. Por lo anterior, es importante indagar en las atenciones en salud realizadas a los pacientes con TB, si tienen algún familiar TB o que tuvo la enfermedad, para detectar nuevos casos y cortar la cadena de transmisión.

La tuberculosis persiste como una de las 10 primeras causas de morbimortalidad en la población mundial. A pesar de que se dispone de un tratamiento efectivo, la TB sigue presentándose a nivel mundial, dado que en muchos casos existen barreras de acceso a los servicios de salud para un diagnóstico y tratamiento precoz, lo cual lleva a las personas portadoras a estados crónicos o bacilíferos, donde se pueden llegar a contagiar de 10 a 15 individuos en su grupo familiar y laboral, que son los contactos cercanos a la persona portadora de la TB 15.

Por lo descrito anteriormente, en Colombia es evidente la limitación de programas de promoción de salud en el lugar de trabajo, especialmente las amas de casa y los mayores de 65 años, en cuanto a las acciones de protección contra la TB. No se ha visto la necesidad de incluir a esta población laboral como un grupo que se encuentra con alto riesgo de contraer enfermedades laborales de este tipo, dada la actividad que desempeñan 16.

En el estudio realizado se encontró que los hombres son más afectados por tuberculosis que las mujeres; la distribución etárea presentó un aumento significativo en dos grupos de 20 a 30 años y mayores de 65 años; el 90 % de los positivos fueron indígenas o migrantes, con un incremento significativo de la cantidad de migrantes. La ocupación fue la variable que mayormente explica la presentación de los casos, y entre ellas las amas de casa y una categoría que se llama "no aplica" son las más importantes (48 %). También hay un incremento significativo de los pacientes que no tienen seguridad social, y no hay diferencias en la forma de diagnóstico en todos los evaluados. Se puede concluir que el hecho de que haya más migrantes, personas sin seguridad social, contribuye al aumento de los casos de tuberculosis observado ♦
